# Responses to Cadmium in Early-Diverging Streptophytes (Charophytes and Bryophytes): Current Views and Potential Applications

**DOI:** 10.3390/plants10040770

**Published:** 2021-04-14

**Authors:** Erika Bellini, Camilla Betti, Luigi Sanità di Toppi

**Affiliations:** 1 Department of Biology, University of Pisa, 56126 Pisa, Italy; erika.bellini@biologia.unipi.it (E.B.); luigi.sanita@unipi.it (L.S.d.T.); 2 Department of Medicine, University of Perugia, 06132 Perugia, Italy

**Keywords:** biomonitoring, bryophytes, charophytes, glutathione, heavy metals, phytochelatins, phytoremediation

## Abstract

Several transition metals are essential for plant growth and development, as they are involved in various fundamental metabolic functions. By contrast, cadmium (Cd) is a metal that can prove extremely toxic for plants and other organisms in a dose-dependent manner. Charophytes and bryophytes are early-diverging streptophytes widely employed for biomonitoring purposes, as they are able to cope with high concentrations of toxic metal(loid)s without showing any apparent heavy damage. In this review, we will deal with different mechanisms that charophytes and bryophytes have evolved to respond to Cd at a cellular level. Particular attention will be addressed to strategies involving Cd vacuolar sequestration and cell wall immobilization, focusing on specific mechanisms that help achieve detoxification. Understanding the effects of metal(loid) pollution and accumulation on the morpho-physiological traits of charophytes and bryophytes can be in fact fundamental for optimizing their use as phytomonitors and/or phytoremediators.

## 1. Introduction

Transition metals are chemical elements of both anthropogenic and natural origin [1] that are widely spread in all environmental matrices (soil, water, atmosphere). Some, such as zinc (Zn), copper (Cu), and iron (Fe) are essential for plants, as for all living organisms [1,2,3,4]. Others, such as cadmium (Cd), mercury (Hg), lead (Pb), etc., as well as the metalloid arsenic (As), are non-essential, as they have no biological functions in all organisms and can be highly toxic for plants, even at very low concentrations [5]. These metal(loid)s are extremely hard to remove, as they are able to penetrate membranes, remain inside cells, and accumulate in tissues and organs. Some are considered highly dangerous pollutants, even with carcinogenic potential for humans; as a result, their levels need to be constantly monitored in the environment and throughout the whole food chain.

Based on their coordination chemistry, metal(loid)s can be classified in three categories, according to their binding preferences: class (A) oxygen-seeking ions, e.g., Li(II), Na(II), Mg(II), K(I), and Ca(II); class (B) nitrogen/sulfur-seeking ions, e.g., Cu(I), Pb(IV), and Ag(I), which show preference for S-containing ligands respect to N-containing ones; and borderline class ions, including ions with intermediate affinity [6]. Metal(loid)s falling into the latter group (As, Cd, cobalt (Co), hexavalent chromium (CrVI), divalent copper (CuII), nickel (Ni), tin (Sn) and zinc (Zn)) show similar preferences for binding O-, S-, or N-containing ligands and thus may represent an overall severe problem for all organisms [1]. Metal(loid)s can also be classified in a different way. According to the Pearson classification, Cr, Cu, and Ni are on the borderline of polarizable and non-polarizable metals, while Cd belongs to the polarizable or soft category, because it is more prone to bind soft ligands, e.g., amino and sulfhydryl groups, with a preference for S-containing ligands [7]. Cr, Cu, and Ni show instead a higher tendency to form stable complexes with hard ligands, e.g., hydroxyl, carboxylate, carbonate, and phosphate groups [8].

Cd is naturally present at low level in the Earth’s crust, where it can form the so-called greenockite, which is a rare Cd-bearing sulfide mineral. Due to the very low presence of greenockite, Cd concentration in the vast majority of non-polluted soils usually ranges around 0.1–2.0 ppm, being mostly below 1 ppm [9]. However, Cd is constantly released into the environment by anthropogenic emissions, given by power stations, heating systems, smelting, urban traffic, application of biosolids, etc., and at times also as a by-product of certain fertilizers [10]. According to the US Environmental Protection Agency (EPA), this makes Cd the third major metal contaminant, after Hg and Pb, representing a threat to the environment [11]. Cd is a metal that poses health risks both to humans and animals, mostly due to food intake, at concentrations found in plant tissues that are generally non-phytotoxic ([12]; FAO/WHO, 2001; UNEP, 2008). For this reason, considerable scientific literature has been produced thus far on Cd toxicity and on Cd resistance mechanisms occurring in higher plants, particularly in crops [10,13,14,15]. Moreover, due to their solubility, Cd and Cd-containing compounds are more mobile than other metals in environmental matrices, possess a good bioavailability, and, consequently, they are generally easily acquired by plants, starting from early plants (charophytes and bryophytes) up to crops (e.g., rice) [12,16]. Cd has been reported to interfere with crucial physiological processes, such as mineral uptake and transport, calcium metabolism, photosynthesis, and respiration [17,18,19,20,21,22].

As Cd can cause severe damage to cell structures and organelles, plants have evolved different response mechanisms to Cd impact. In fact, once Cd has passed the cell wall, it enters the cytoplasm and follows several, not mutually exclusive, pathways: (i) it can be extruded out of the plasma membrane via transporters, as the *Arabidopsis* ATP binding cassette (ABC) transporter Pleiotropic Drug Resistance 8 (AtPDR8) [23]; (ii) it can be chelated with thiol-containing compounds such as glutathione (GSH) and/or phytochelatins (PCn) and then transported into vacuoles via ABC-like transporters [24]; (iii) it can act as an indirect oxidative stress modulator, thereby affecting the activity of the enzymes superoxide dismutase (SOD), ascorbate peroxidase (APX), catalase (CAT), and peroxidase (POD), thus resulting in the production of reactive oxygen species (ROS) that can cause DNA damage, lipid peroxidation, protein modifications and disruption of cellular membranes [25,26,27,28,29].

Most organisms, and especially plants (given that they are sessile), have evolved specific mechanisms for regulating metal accumulation, in order to preserve their health and possibly achieve tolerance [30]. In particular, some charophytes and bryophytes (also named “early-diverging streptophytes”) appear to have a greater ability than other plants to grow and survive in substrates containing high Cd concentrations [16,31]. This feature makes these early plants optimal organisms for studying morpho-functional traits/mechanisms underlying resistance to Cd toxicity, i.e., specific anatomical (e.g., higher surface/volume ratio) and/or biochemical (e.g., more efficient Cd chelator enzymes or molecules) traits. A further characterization of these plants could also be exploited in phytoremediation approaches (e.g., a higher bioaccumulation factor for defined species). A detailed overview of the anatomical and functional features of bryophytes and charophytes that make this strategy possible will be presented herewith.

Early-diverging streptophytes are commonly used to study the evolution of important biological mechanisms such as plant resistance to metal pollution, because they represent the evolutionary connection between aquatic and terrestrial life [16]. Charophytes (Charophyta) are a paraphyletic group of green algae sharing several biochemical, molecular, physiological, and ultrastructural similarities with land plants [32,33,34,35]. As charophytes are a sister group of all land plants [36,37], they can provide important “primeval” information, which is fundamental for reconstructing several biological functions from an evolutionary point of view [33,38,39]. Moreover, charophytes are still closely linked to the aquatic environment, where metal micronutrients often represent a growth-limiting factor [40]. This makes them excellent model organisms for understanding the evolution of metal regulation and the mechanisms behind metal toxicity responses.

Bryophytes (liverworts (Marchantiophyta), mosses (Bryophyta), and hornworts (Anthocerotophyta)) are considered the earliest-diverged lineage of land plants [41]. Concerning their phylogeny, until recently, the accepted hypothesis was that they are paraphyletic, with liverworts, mosses, and hornworts being successive sister lineages to tracheophytes [42]. However, there is now mounting evidence that bryophytes are monophyletic, with hornworts sister to mosses and liverworts [43,44]. Bryophytes are important in studies concerning the evolution of metal detoxification systems during the transition from water to land. Not least, bryophytes possess a very high surface/volume ratio, have an elevated cation exchange capacity, do not develop strong hydrophobic barriers and, consequently, are prone to “uncontrolled” metal absorption [45,46,47,48]. Therefore, due also to their wide geographical distribution, bryophytes have been used as an important biological monitoring system for metal pollution [49].

This review deals with functional and metabolic aspects, and related responses, of charophytes and bryophytes to Cd stress. The valence of such early-diverging streptophytes as phytomonitors and phytoremediators is also discussed.

## 2. Cadmium Effects on Charophytes and Bryophytes

Cd is a toxic transition metal, due to its negative effects on plants’ health, at a morphological, (ultra)structural, molecular, and functional level. Its competition with other divalent metals may result into binding ligands that may also bind other metals [15]. These ligands are often cysteine (Cys), histidine (His), and/or other amino acids, as they are common both in a free form and in various enzyme metal centers, in particular in those containing Zn [50]. Since Cd^2+^ ions can replace Zn^2+^ due to their chemical similarities, therefore, Zn-dependent and Zn-binding molecules represent potential targets of Cd^2+^ toxicity. Since Zn often has a structural role in enzyme conformation, as in the case of Cu/Zn-SOD, Cd-substituted enzymes are usually not active because of the derived conformation changes [51,52]. Furthermore, the chemical similarity to Zn^2+^ often implies that Cd^2+^ uptake and distribution exploit the same transporters as Zn^2+^ ions [9,17]. The direct competition for transporters can also reduce the uptake of other essential elements, causing nutritional deficiency phenomena or alterations in metal distribution pathways [18]. There is, in fact, evidence indicating that Ca^2+^ channels [19] and Fe^2+/3+^ transporters can also facilitate Cd^2+^ uptake [53]. The aforementioned aspects have been explored in detail only in higher plants, but it is legitimate to hypothesize their extension also to charophytes and bryophytes, at least in conceptual terms.

The photosynthetic apparatus is also negatively affected by Cd exposure, particularly in early-diverging streptophytes. In fact, it is well known that Cd^2+^ can replace Mg^2+^ both in the RuBisCo catalytic center and in chlorophyll (Chl) porphyrin ring [15]. In the latter, Cd binding induces bleaching and consequent degradation, thereby causing decreases in photosynthetic activity, which results in serious impairments [54]. There are few studies on the [Cd]-Chl bond formation because of the high similarity between [Cd]- and [Mg]-Chl UV/VIS absorption spectra, which makes it difficult to interpret the results. Moreover, the instability of the [Cd]-Chl binding causes high degradation rates during the extraction/separation phases [54]. A reduction in the total Chl content as a specific response to metal stress has been measured in early-diverging streptophytes, with different degrees depending on the species and the metals tested ([16], and reference therein). In particular, in the moss *Physcomitrium* (*Physcomitrella*) *patens*, a decline in Chl content upon Cd exposure has been observed, also causing a subsequent loss in cell viability [55]. Similar results have been obtained in the aquatic moss *Fontinalis antipyretica*, which showed a significant decrease of Chl content after exposure to increasing concentrations of Cd, while no differences were measured after treatments with Pb [56]. Variations in the reduction of Chl content upon metal exposure can be explained by their specific uptake and action mechanisms [47,57]. Likewise, in the liverwort *Marchantia polymorpha*, Cd is the only metal amongst Cd, Pb, Cu, and Zn that significantly affects Chl content, whereas it does not affect carotenoid content [58]. Evidence worth noting is that unlike bryophytes, the negative effects of Cd exposure on Chl content seem not to occur in charophytes. In fact, Clabeaux and colleagues [59] demonstrated that Chl *a* and *b* levels were not affected in *Chara australis* even after Cd treatments at growth-suppressing concentrations. Nevertheless, a few studies have been conducted on charophytes, and it would be necessary to deepen the knowledge of these plants.

Alterations of nitrogen metabolism are also important effects induced by Cd [60]. For instance, the moss *F. antipyretica* grown in the presence of increasing concentrations of Cd shows a concentration-dependent decrease of nitrogen incorporation into amino acids. This is due both to the lowered nitrogen uptake caused by plasma membrane damage and to a concentration-dependent inhibition of protein synthesis [60]. In general, plasma membrane damage is consequent to lipid peroxidation phenomena that cause membrane peroxidation due to the massive amounts of ROS produced and their derived incomplete detoxification [61]. Although Cd is not a redox active metal per se, its presence can, in fact, lead to an indirect overproduction of reactive oxygen species (ROS) [28]. One likely reason of this induction is the enhanced mis-transfer of electrons to oxygen instead of to the target molecule, e.g., in the above-mentioned formation of the [Cd]-Chl bond. Another possible reason is that Cd^2+^ exposure reduces the capability of ROS scavenging. The ROS steady-state levels are tightly regulated by the interplay between different ROS-producing and ROS-scavenging mechanisms. ROS-scavenging mechanisms depend on the activity of both non-enzymatic antioxidants, such as GSH and ascorbate, and redox enzymes, such as SOD, CAT, and the other enzymes sustaining the Halliwell–Asada cycle; Cd treatment can alter their synthesis or activity, thus leading to oxidative stress in plant cells [28]. Moreover, the effect of metals on lipid peroxidation and membrane distortion mediated by ROS can also be investigated by analysing the intracellular malondialdehyde (MDA) content. MDA is a cytotoxic product of lipid peroxidation, and an increase in MDA and ROS content has been observed in *M. polymorpha* exposed to Cd, while a general absence of oxidative stress was detected for Pb and Zn treatments [58]. Furthermore, in the moss *Leptodictyum riparium*, an induction of oxidative stress, measured in terms of a significant increase in ROS production, proportional to the Cd concentration used for treatments, has been observed [62].

Along with biochemical and physiological alterations, Cd exposure also causes specific ultrastructural changes in bryophyte and charophyte anatomy and (ultra)structure. In particular, dose-dependent ultrastructural modifications have been observed in the moss *Scorpiurium circinatum* exposed to Cd and Pb, while Cu- and Zn-treated samples showed similar anatomical alterations regardless of increasing metal concentrations [63]. In bryophytes, the shape of chloroplasts and thylakoid membrane arrangement are generally affected by toxic substances—in particular by Cd [64,65]. In fact, in *S. circinatum*, an altered chloroplasts shape, disorganized thylakoids, and an increased number of plastoglobules have been observed under Cd exposure, both in laboratory and environmental polluted conditions [63,66]. Plastoglobules are directly involved in the synthesis and storage of tocopherols, which protect membrane lipids from photo-oxidation, and photosystem II from photo-inactivation [67,68]. Thus, under oxidative conditions, tocopherols stored in plastoglobules are delivered to thylakoid membranes to scavenge ROS [69]. In addition, in some cases (i.e., in *L. riparium*), plasmolyzed cells showing cytoplasm vacuolization have been found [62,70]. This might be a consequence of the loss of membrane-selective permeability, arising primarily from direct membrane damage or, secondarily, from cellular energy depletion [71]. Other peculiar intracellular alterations, such as the presence of multivesicular bodies and autophagosome formation, have been reported in the charophyte *Micrasterias denticulata* [72,73], in the freshwater moss *L. riparium* [74], in the liverwort *Lunularia cruciata* [75], and in other bryophytes [63,65,76]. Similar ultrastructural changes have been also reported in other charophytes, such as in *Nitella mucronata*, where extensive symptoms of toxicity have been observed in the presence of 36 µM Cd, whereas no specific alterations in cell ultrastructure were seen after Zn treatment [77]. The effects of Cd on charophytes and bryophytes are summarized in Table 1.

## 3. Response to Cadmium Toxicity in Charophytes and Bryophytes

Plant cells have developed different strategies to cope with Cd stress and to limit its toxic effects, which are collectively known as the “fan-shaped response” [10]. This complex phenomenon includes various mechanisms that might come into play in response to Cd, both in an additive and in a mutually potentiating way. The first line of defense aims to avoid metal uptake, considering that Cd toxic effects are chiefly caused by its intracellular fraction. The avoidance mechanism of Cd intracellular accumulation includes all the processes preventing the entrance of the metal into the protoplast [78]. This strategy is predominant in bryophytes, which are characterized by a high surface/volume ratio that makes them highly effective in chelating cations on their surface, as the absorption involves the whole gametophyte. In particular, free Cd^2+^ ions may interact with the negatively charged cell wall [48,59].

In charophytes, in addition to the metal binding to the cell wall, a high mucilage production, induced as a stress reaction to protect the plant, also seems to play a key role in metal intake [79,80]. In fact, mucilage appears to be a metal bind site via calcite, as seen with uranyl species binding to the cell walls of *Chara fragilis* [81]. The adsorbed metal can be trapped as particulate matter within the surface layer bound either to the cell wall or to the outer surface of the plasma membrane.

The extracellular accumulation of metals is mediated by an ion exchange process [82] and the formation of complexes between the metals and the functional groups occurs in the cell walls of bryophytes [47]. In this mechanism, the cell wall plays a key role, and its binding capacity is clearly related to its composition [70,83]. In fact, differences in the cell wall chemical composition between mosses and liverworts, or between different species in the same group, could explain the differences in Cd uptake and, thus, the differences in their sensitivity to this pollutant [47]. In particular, in bryophytes, the high metal binding capacities are often ascribed to uronic acids, which are typical components of their cell wall, together with mannose-containing hemicellulose and 3-O-methyl rhamnose [84]. Similarly, charophyte cell walls also contain 3-O-methyl rhamnose, together with high amounts of mannose-containing hemicellulose, glucuronic acid and mannuronic acid [85]. Moreover, early-diverging streptophytes do not contain lignin or cutin, despite the presence of lignans and other lignin-like polymers [84]. Thus, the abundant ion-exchange sites are responsible for the high biosorption capacity of early diverging land plants, as demonstrated in the moss *S. circinatum*, where the cell wall is the main detoxification site capable of immobilizing Cd ions [63]. Moreover, there are different studies describing a metal avoidance mechanism, where binding of the metal ion to the cell wall reduces the amount of metals entering the protoplasm of mosses [66,86,87]. The cell wall system of mosses seems to be, in fact, an efficient barrier to metals, and particularly Cd, not only in gametophytes but also in sporophytes, in which a higher metal adsorption at cell wall level has been measured [78,88,89]. In addition, Basile et al. [90,91] observed that metal ions were sequestered inside the placenta of the moss *Funaria hygrometrica* by cell wall labyrinths, preserving the above sporophyte from being damaged by the metals. Furthermore, samples whose placenta had completely degenerated did not show a significant difference in metal concentration between the two generations, since the degeneration of the placenta allows water and solutes to pass directly from the former to the latter, indicating the absence of the protective role of the placenta [90].

**Figure 1:**
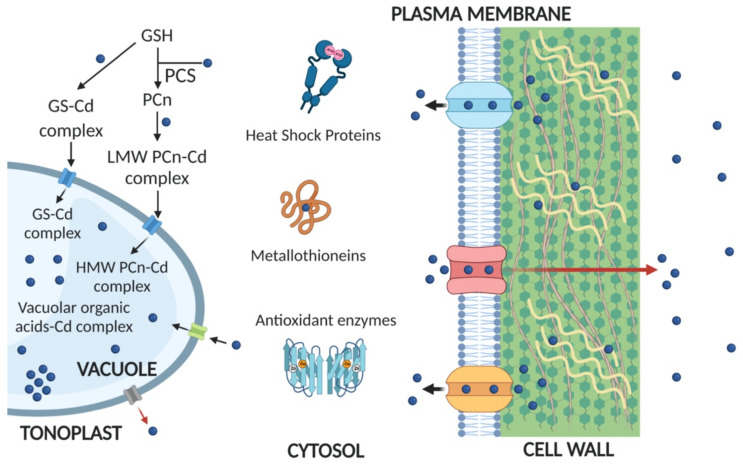
Cd transport and detoxification mechanisms in early-diverging streptophytes. Blue circles represent Cd. Red arrow shows a transporter-mediated Cd extrusion mechanism via transporters such as CDF (Cation Diffusion Facilitators) or PDR (ATP Binding Cassettes), whereas black arrows depict Cd internalization via different transporters such as NRAMP (Natural Resistance-Associated Macrophage Protein) or IRT (Iron-Regulated Transporter, ZIP family). Tonoplast transporters in blue represent systems responsible for Cd uptake into vacuole, such as HMA (Heavy Metal-Associated) or MRP (ATP Binding Cassettes). Tonoplast transporters in gray represent NRAMPs, which are responsible for Cd efflux out of the vacuole. GSH: reduced glutathione; GS-Cd: glutathione-bound Cd; PCn: phytochelatins; PCS: phytochelatin synthase; PCn-Cd: phytochelatin-bound Cd; Vacuolar organic acids-Cd complex: complex formed by Cd binding to Krebs acids and derivatives; LMW and HMW: Low and High Molecular Weight. Created with BioRender.com.

Despite these avoidance mechanisms, Cd resistance may be also achieved by restricting Cd influx at the plasma membrane and/or by eliminating Cd by extruding it out from the plasma membrane (Figure 1) [81]. Nevertheless, the plasma membrane transporters involved in the extrusion of free Cd ions, or Cd conjugates, has yet to be identified in early-diverging streptophytes. Future studies could focus on the functional characterization of putative orthologues of Cd transporters showing high homology with Cd extrusion systems already identified in higher plants, such as the ABC transporter AtPDR8 in *A. thaliana* [23].

Once Cd has arrived in the cytosol, a series of detoxifying pathways can be activated, such as the neutralization of free ions by their compartmentalization in vacuoles and/or by detoxification mechanisms mediated by metal chelation. In the latter case, it has been widely demonstrated in higher plants that the free Cd can be detoxified by ligands, such as the thiol-containing tripeptide glutathione (GSH) and the oligomers of GSH, which are called phytochelatins (PCn) [9,10]. PCn are thiol-oligopeptides whose general structure is (γ-glutamate–cysteine)n–glycine, with n usually ranging from 2 to 5 [92]. Due to the thiol group of Cys residues, PCn can bind Cd and other thiophilic metals and prevent them from circulating in the cytosol [92]. PCn are synthesized from GSH by means of the constitutively expressed cytosolic enzyme phytochelatin synthase (PCS), which is a γ-glutamylcysteine dipeptidyl (trans)peptidase (EC 2.3.2.15) [93,94], belonging to clan CA of the papain-like Cys proteases [5,95,96]. The transpeptidasic activity of PCS is displayed when the enzyme’s catalytic site tightly binds complexes between GSH or its direct thiol-derivatives, and metal(loid)s, such as Cd, Pb, Hg, As, Cu, Zn, and iron (Fe). In particular, the PCS activation is self-regulated, because PCn chelate Cd, and the reaction ceases when free Cd ions are no longer available [97]. Once PCn have been synthesized, they may rapidly form “low molecular weight” (LMW) complexes with Cd [10]. LMW complexes can acquire acid–labile sulfur (S^2−^), probably at the tonoplast level, to form “high molecular weight” (HMW) complexes [98], which have a higher affinity toward Cd ions (Figure 1). Then, HMW complexes are accumulated in the vacuole, where they dissociate because of the acidic pH, thereby releasing Cd, which can be complexed by vacuolar organic acids (e.g., citrate, oxalate, malate) and/or, possibly, by amino acids [99]. Apo-PCn (complexes without Cd ions) may be degraded by vacuolar hydrolases and/or return to the cytosol, where they can continue to carry out their shuttling role [10]. Although it was thought for long time that bryophytes do not synthesize PCn under metal stress [55,57,100,101,102], Petraglia et al. [103] demonstrated instead that constitutively expressed and functional PCSs are present in a number of bryophytes, as well as in other early-diverging streptophytes, arguing that the ability to synthesize PCn, as well as the presence of active PCSs, are ancestral (plesiomorphic) traits of early-diverging plants [103]. This study was followed by others, supporting the idea that PCn synthesis is an important mechanism of intracellular Cd detoxification, both in bryophytes, such as in *L. riparium* [62], *L. cruciata* [75,104], *M. polymorpha* [105,106], and in charophytes, such as *N. mucronata* [77]. In addition to the ability to synthesize PCn, vacuolar compartmentalization plays a key role in metal detoxification in bryophytes and charophytes, as well as in higher plants, as supported by the intravacuolar electron-dense Cd deposit detected in *L. cruciata* [75]. Moreover, this study also demonstrated that phosphate could be involved in Cd vacuolar sequestration, probably by forming insoluble Cd–phosphate complexes, as shown by the apparent increase in P and O X-ray peaks detected in Cd-exposed gametophytes [75].

Interestingly, some bryophyte species seem to lack the PCS gene, including the model-moss *P. patens*, which has an entirely sequenced genome [107,108]. In this species, the gene-encoded metallothioneins (MTs) appear to play a key role in the intracellular chelation of Cd (Figure 1), since four MT-like genes have been identified in its genome, three of which were shown to provide Cd resistance when expressed in yeast [109]. MTs are low-molecular-weight Cys-rich proteins ubiquitously found in nearly all eukaryotic organisms and some bacteria [110]. The Cys residues are arranged in the metal-binding motifs Cys–Cys, Cys–X–Cys, or Cys–X–X–Cys, and their positions are conserved within a specific group of related MTs [111]. Noteworthy, plant MTs (pMTs) are much more diversified in terms of primary structure with respect to MTs from other organisms. In fact, in pMTs, a significantly higher degree of heterogeneity in terms of primary sequence length, amino acid composition, and cysteine number and arrangement is found [112]. Four discrete topologies of the cysteine patterns recurring in pMTs can be categorized, namely Types 1 to 4 [111,113]. The length of cysteine-free linkers between the cysteine-rich regions is different in each group, and this very likely reflects important changes in the final three-dimensional protein structure and related function. This allows pMTs to carry out different physiological roles, as also supported by a wealth of evidence indicating that some pMTs show constitutive expression, while some others can be induced by endogenous and/or exogenous signals, both following specific spatial and temporal regulations [113]. In general, the sulfhydryl ligands of Cys are known to participate in high-affinity coordination of multiple metal ions in different species, e.g., up to four Cd ions in the α-metal-binding domain and three Cd ions in the β-domain of rat MT-2 [114]. MTs are generally believed to act in essential micronutrient homeostasis and metal detoxification, although these roles have only been demonstrated by genetic evidence in model organisms, as in a pioneering study conducted in 1986 on *Saccharomyces cerevisiae* [115]. Recent studies on pMT isoforms in *A. thaliana* knockout lines suggested that MTs may have little or no direct contribution toward metal detoxification [116,117]. However, other results demonstrated a role for pMTs in ROS scavenging and regulation, which are pivotal mechanisms in plant response to oxidative stress [118,119,120] and/or in plant development [121,122,123]. These different functions could be explained by their primary structure heterogeneity, as aforementioned. Despite the typical pMTs aminoacidic features and their wide distribution, not much is known about pMTs of non-vascular plants. To date, pMT sequences were found only in the mosses *P. patens*, *Grimmia pilifera*, and *Syntrichia ruralis*, as well as the liverwort *M. polymorpha* [113]. Moreover, it appears that bryophytes contain genes encoding only for Type 1 MTs, suggesting that this group might be the most primitive one [109]. However, despite bryophytic MTs belong to Type 1, they still show substantial internal heterogeneity on cysteine number and arrangement. For example, two of the four putative MT genes identified in bryophytes are considered to be a unique sub-type (1.2), as they could be distinguished by the presence of a Cys–Cys–Cys motif in the N-terminal Cys-rich domain, in place of the Cys–X–Cys motif in the other MTs [109]. Therefore, it would be important to deepen the knowledge about the structure and the role of these proteins also in early-diverging streptophytes, in order to better clarify their functional significance throughout the evolutionary history of plants.

Noteworthy, Krebs acids and derivatives (citrate, malate, oxalate, etc.) are also metal chelators representing a further possibility of the intracellular responses to metal-induced stress in tracheophytes, where metal–organic acid complexes can be formed in the vacuole [124]. Although not extensively studied in non-vascular plants, Krebs acids have recently been monitored and identified as one of the players contributing to Cd detoxification in mosses [125,126]. In particular, malic acid has been shown to contribute to Cd chelation in the mosses *Taxiphyllum barbieri* [125] and *P. patens* in which also citric acid plays a role in Cd detoxification [126].

As previously mentioned, Cd ions generate oxidative pressure in the plant cell, and subsequent toxicity. In this context, the intervention of a series of antioxidant mechanisms is also observed in bryophytes, such as the variations in SOD, CAT, and GST activities that are linked to Cd stress [62,104]. Interestingly, the GST enzyme plays a dual role in Cd response, because it can simultaneously counteract the oxidative stress by enhancing ROS quenching and detoxify a number of electrophilic xenobiotics or chemical elements, including Cd [127,128,129]. In fact, GST contributes, together with the above-mentioned mechanisms, to the vacuolar compartmentalization of Cd. In particular, GST catalyzes an intracellular detoxification reaction of metals or other noxious compounds by forming a cytosolic conjugate between GSH and the toxic element/substance, which is followed by the sequestration of this conjugate (GS-conjugate) in the vacuolar compartment [130,131] (Figure 1). In bryophytes, metal detoxification by conjugation and the subsequent translocation of the conjugate into the vacuole was visualized in fluorescence microscopy by providing monochlorobimane (MCB) to Cd-exposed gametophytes of the moss *L. riparium* [62].

As one of the toxic effects of intracellular Cd is the induction of protein denaturation, bryophyte response to Cd also includes the induction of intracellular proteins involved in protein refolding in response to stress, e.g., chaperones. Heat-Shock Proteins (HSPs), such as HSP70, have been reported to increase after Cd treatment in the liverwort *Conocephalum conicum*, confirming that protein refolding is necessary to maintain cellular activity in the presence of metal stress [74,76,104,132]. The same intracellular detoxification mechanism has been also described in higher plants [9,10], and reference therein.

Among the multiple strategies adopted to prevent Cd intoxication, there are also morpho-physiological mechanisms linked primarily to metal allocation in the whole gametophyte. For example, in *L. cruciata* gametophytes, it has been demonstrated that water solutions are conveyed by the nerve and distributed to the wings through the vacuolated photosynthetic tissue and the highly vacuolated hyaline parenchyma [65,133]. The preferential allocation of Cd into the edge of the wing—rather than the nerve—may reflect the occurrence of a mechanism preventing intoxication of the apical tissues [65,75,76]. The different responses to Cd toxicity in early-diverging streptophytes are summarized in Table 2.

## 4. Charophytes and Bryophytes: Application Potential

The need to monitor the presence, concentration, and distribution of metal(loid)s in the environment, especially Cd, originates from their toxicity to all organisms, particularly to humans. These pollutants show a high persistence in ecosystems, due to their average fair solubility (thanks to which they are readily absorbed by plants) and ability to accumulate in organisms in concentrations higher than those found in the surrounding environment [10,134]. In fact, the use of various organisms in environmental quality studies has been strongly recommended for many years now, in order to obtain corroborative information that integrates chemical and physical data [135,136]. The use of biological systems capable of absorbing metal(loid)s in such a way that their tissue loads reflect the concentrations in the environment, and their distance from the sources, can give quantitative information about metal(loid) pollution in all environmental matrices and account for its effects on the biosphere [137,138]. Biological responses can be considered more representative than data supplied by chemical or physical detectors, as they are spatially and temporally contained; moreover, they allow estimating both the levels of pollutants and, even more importantly, the impact on biological receptors. Thus, analyses on organisms collected from polluted sites are fundamental to obtain quantitative data about the presence of each specific metal, including Cd [139].

Amongst all organisms, photoautotrophs can act as optimal bioindicators, biomonitors, and bioaccumulators, and they display a high resistance to persistent pollutants in general and to metals in particular [140,141,142]. With regard to plants, bryophytes, and specifically mosses, are more frequently used as phytomonitors than other plants because of their great ability to retain high concentrations of metals, which are both airborne and/or from soil and aquatic environments [143,144]. In addition, bryophytes are highly recommended in large-scale surveys due to their peculiar morpho-anatomical structures, accumulation mechanisms, and ecophysiology [145,146]. The use of bryophytes is recommended in long-term studies because they are poikilohydric perennial plants, enabling sampling throughout the year, with a morphology not varying with seasons. Thus, their bioaccumulation can continuously occur over time [146,147]. It is worth noting that due to the absence of specialized conducting tissue [137] and the slow growth rate [138], bryophytes can provide data about integrated exposure to metals over longer periods of time, and not just about a current state, which is particularly important in areas where levels of introduced metals change rapidly.

Furthermore, given that bryophytes were the first plants to permanently colonize the terrestrial environment starting from the Ordovician–Silurian periods [148], they had to develop mechanisms to cope with metals present in far greater amounts in the (paleo)soils than in water [75]. Therefore, millions of years of evolution have created, on one hand, bryophyte species that can accumulate large amounts of metals in extremely polluted areas without any visible negative effect on their growth and development [149,150]; and on the other hand, species that are susceptible to pollution and reflect visible symptoms of damage, even in the presence of low concentration of pollutants, and may therefore serve as good bioindicators of the degree of environmental pollution [137]. In the first group, specifically used for phytoaccumulation studies, many mosses are found (for a detailed list of moss species for biomonitoring, refer to [147]), although also some liverworts [65,83] have recently proven useful for this application, thanks to their morpho-physiological properties [151]. In general, bryophytes lack a root system, as they only have root-like filaments (rhizoids) that anchor them to their substrates, i.e., tree bark, soil, rock, or sand [152]. Thus, due to the rhizoid system, bryophytes largely depend upon atmospheric deposition to fulfill their nutrient requirement. Moreover, they have a high surface-to-volume ratio compared to tracheophytes, thereby helping to improve their absorption ability [153,154]. This is also facilitated by the presence of just a very thin layer of cuticle, or even no cuticle, over their epidermis [155], which makes their tissues readily permeable to water, gaseous pollutants, and several metal(loid) ions [45,46]. In addition, their pronounced ion-exchange properties significantly contribute to this ability [156]. Consequently, some bryophytes act as the perfect basin for the deposition of environmental pollutants, as discussed earlier, and can react to, and reflect, changes in the metal(loid) concentrations faster than most tracheophytes (Table 3) [157]. Nevertheless, the correlation between the concentration of metals in soil and the real amount found in plant tissues is often very difficult to establish, making dose-related studies somewhat unwieldy [16]. Thus, the importance of performing in vitro evaluations of metal(loid) uptake, starting from known concentrations, is of crucial importance in order to unravel the real metal(loid)-scavenging capacities of these early plants (e.g., the maximum absorption load that the plant can accommodate without encountering any morphological and physiological toxicity symptoms) (Table 3).

Charophytes, on the other hand, have been little used for biomonitoring purposes to date, despite their clear potential as biosorbents, as they are ubiquitous and abundant in aquatic environments, on average have a fast growth rate, are easy to harvest, and have a good affinity for a variety of metal(loid) ions (Table 3) [158,159]. *Chara* and *Nitella* are the main genera of the characean green algae (Characeae) growing abundantly in a wide range of fresh and brackish water bodies [38,160]. In eutrophic areas, the abundance of some *Chara* and *Nitella* species may reach a nuisance level, reduce other plant and animal diversity, require disposal via manpower or biological and chemical control, and produce a regenerative source as a large-scale biomaterial [161,162]. This biomaterial can be used for the removal of metals, including Cd, from wastewater or contaminated water bodies either by biosorption or by bioaccumulation [163,164,165], which involve interactions and concentration of toxic metals or organic pollutants within the biomass, either living (bioaccumulation) or non-living (biosorption) [166]. In fact, some species (such as *Nitella graciliformis*, *Chara aculeolata*, and *Nitella opaca*) demonstrated their ability to take up metals from ambient solutions and accumulate them in the plant tissues at high concentrations [31,164]. Furthermore, the use of dried dead biomass, as in the case of *C. aculeolata* and *N. opaca*, represents another prominent application displaying high potential for biosorption of Pb, Zn, and Cd for treatment of multi-metal solutions [167]. Crucially, their anatomy is functionally not too far from that of higher plants. In fact, they have rhizoids, fine filaments imbedded in the sediment, which, similar to roots, anchor the plant and have some role in the uptake of nutrients. The aboveground cauloids can import nutrients from the rhizoids or accumulate them directly from the water column. In addition, rhizoids can also regenerate cauloids, facilitating continuous, sustainable harvest. The charophyte-mediated bioaccumulation capacity also relies on their ability to calcify, which enables them to bind metals via calcite, as shown with uranyl species binding to the cell walls in *Chara fragilis* [81]. The alga *Chara australis* has a high ability to resist and accumulate Cd in both cauloids and rhizoids, and it can accordingly be used for the remediation of contaminated sediments [168]. As a result, this biomineralization potential of charophytes can be very useful in phytoremediation. Not least, some species of the genus *Chara*, as *C. australis*, are also considered as likely candidates for the phytoextraction of Cd in contaminated sediments [59]. A qualitative comparison of advantages and disadvantages of the use of early-diverging streptophytes in phytomonitoring and phytoremediation approaches is represented in Table 3.

## 5. Conclusions

In conclusion, the mechanisms able to detoxify metal(loid)s in charophytes and bryophytes certainly deserve more in-depth studies. In particular, the role of GSH requires further experimental investigation, considering its high abundance, on average, in these early-diverging streptophytes [57,62,103]. It should also be mentioned that PCS and PCn have recently been detected in several bryophytes and charophytes, suggesting that the role of PCn in the control of metal detoxification and homeostasis can be relevant in these plants, too [77,103]. More detailed studies on such organisms, which hold a key position in phylogenesis, might reveal the presence of other important detoxification mechanisms that have been lost over evolution and/or better clarify the molecular mechanisms behind the high resistance to metal(loid)s evidenced by these plants. Studies on the players involved in achieving resistance to high Cd concentrations, peculiar only to bryophytes, could help achieve a better understanding of the evolution of defense mechanisms to abiotic stress from water to land plants. Not least, given the highly variable conditions encountered during in-field sampling, it is crucial to perform this kind of study also in controlled conditions. Bearing in mind the importance of early-diverging plants in metal(loid) response, it would also be interesting to investigate such strategies in hornworts, which is a group of bryophytes for which there is no information on this specific aspect to date. A deeper look into the past can help to understand the present and find effective strategies to address future challenges.

## Figures and Tables

**Table 1:** Cd effects in charophytes and bryophytes.

Effect	Reference No.
Essential metal ion replacement	[51,52]
Uptake reduction of essential elements	[9,17,18,19,53]
Mg^2+^ replacement in the RuBisCo catalytic centre and in Chl porphyrin ring	[15,54]
Total Chl content reduction	[16,47,55,58,59]
Nitrogen metabolism alteration	[60]
Oxidative stress induction	[28,58,61,62]
Shape of chloroplasts and thylakoid membrane arrangement alterations	[63,64,65,66]
Cell plasmolysis and cytoplasm vacuolization	[62,70]
Multivesicular bodies and autophagosome formation	[63,65,72,73,74,75,76,77]

**Table 2:** Response to Cd toxicity in charophytes and bryophytes.

Response	Reference No.
**Avoidance mechanisms**	
Cd adsorption onto the cell wall	[16,47,48,59,63,66,73,78,81,86,87,88,89]
Cd chelation by the mucilage produced in charophytes	[79,81]
Cd sequestration in the placenta of mosses	[90]
**Detoxification mechanisms**	
Cd chelation by PCn	[62,75,77,103,104,105,106]
Cd chelation by vacuolar organic acids	[124,125,126]
Cd complexation by phosphate	[75]
Antioxidant response	[63,104]
Heat Shock Protein induction	[74,76,104,132]
Metal allocation arrangement	[65,75,76,133]

**Table 3:** Advantages and disadvantages of the utilization of charophytes and bryophytes in metal(loid) phytomonitoring and phytoremediation.

	Phytomonitoring and Phytoremediation	
	**Advantages**	**Disadvantages**
**Charophytes**	Fast growth rate [158,159]	Little known responses in metal(loid) phytomonitoring and phytoremediation
	Easy to harvest [158,159]	Use limited to freshwater wetlands [160]
	High metal(loid) bioaccumulation [163,164,165,168]	
	Both dead (dry) and living biomass can be used [166]	
	**Advantages**	**Disadvantages**
**Bryophytes**	Good performance in bioindication [145,146]	Low growth rate [138]
	High metal(loid) bioaccumulation capacity [146,147,149,150]	Low biomass production for most species [138]
	*Sphagnum* species produce large biomass and may record past pollution events in peat bogs [169,170,171]	
	High surface/volume ratio [153,154]	
	Lack of (or very thin) protective cuticle(true also for charophytes) [155]	
	Use of moss bags in areas where bryophytes are naturally lacking [144]	
	Somatic desiccation tolerance allows some mosses to survive in prolonged exposition to air [172]	
